# Community-based solutions for chronic disease management during natural disasters: A systematic review

**DOI:** 10.1371/journal.pgph.0004997

**Published:** 2025-08-01

**Authors:** Aditi Iyer, Arthur Bookstein, Giselle Kim, Justine Po

**Affiliations:** 1 Department of Neuroscience, Duke University Trinity College of Arts & Sciences, Durham, North Carolina, United States of America; 2 Keck School of Medicine, University of Southern California, Los Angeles, California, United States of America; University of Greenwich, UNITED KINGDOM OF GREAT BRITAIN AND NORTHERN IRELAND

## Abstract

Despite rapid increases in both the burden of chronic disease and climate change-driven extreme weather events globally, the need to jointly address these crises remains largely overlooked. Chronic diseases require ongoing and often specialized care, which natural disasters disrupt by increasing physiological stressors and disrupting access to healthcare facilities, food, shelter and medications. Community-based solutions can mitigate these health risks, especially in low-resource settings and among historically underserved populations. This systematic review aimed to identify key recommendations for community-based interventions that aid with chronic disease management during extreme weather events. A comprehensive search strategy was used to yield 46 eligible studies from 266 search results from Embase, PubMed and Google Scholar. Articles were included if they discussed chronic diseases, community-based solutions and natural disasters and excluded if they were not English-language and/or not published in a peer-reviewed journal. Articles were assessed and selected using PRISMA guidelines, and their quality was assessed using the Joanna Briggs Institute Critical Appraisal Tools. The final sample of studies represented 19 countries and consisted of four quantitative, 35 qualitative and seven mixed-method studies. Minimal quantitative data was a key limitation of this review and topic, which requires further research. From the selected studies, a narrative synthesis approach was used to derive nine themes of solutions. Findings reflected the highest number of interventions and recommendations targeting patient education, continuity of medication management, stakeholder collaboration and digital health.

## Introduction

Non-communicable diseases (NCDs)—chronic conditions caused by genetic, physiological, environmental and behavioral factors rather than infectious agents—are the leading cause of death and disability globally, with rates rising rapidly over the last two decades particularly in low- and middle-income countries (LMICs) [1]. Despite the significant and growing burden of NCDs, public investment in their prevention and control remains disproportionately low, especially when compared to other global health priorities [2,3]. This disparity is especially problematic for historically underserved populations—such as displaced, racial/ethnic minority and low-income communities—who are more vulnerable to the compounded effects of NCDs and natural disasters. In the aftermath of Hurricanes Maria and Irma, 30% of deaths were due to chronic disease complications [4] and between 2007 and 2020, 72.5% of non-accidental deaths attributed to wildfire smoke exposure were linked to chronic conditions [5].

Marginalized communities are not only disproportionately affected by NCDs but also more likely to reside in hazard-prone areas. These communities often lack adequate disaster preparedness education and essential physical resources, such as food, water, stable shelter and temperature control. As a result, 32.6 million people were internally displaced due to disasters in 2022 alone [6]. Moreover, projections suggest that climate-related disasters could double the number of people requiring humanitarian aid, reaching over 200 million annually by 2050 [7]. Even after being displaced, nearly 60% of refugees and internally displaced people continue to live in countries that are highly vulnerable to climate change [8].

In the face of concurrent crises and limited funding, community-based interventions pose a promising solution for chronic disease management (CDM) during extreme weather events. Such interventions empower community members to identify and address locally relevant challenges. Often, they involve multisectoral partnerships that bring communities together with local academic institutions, government bodies and businesses to leverage a diverse range of expertise [9]. Community engagement before and after a disaster has been shown to reduce disaster-induced physical health problems and adverse experiences, decrease the risk of post-traumatic stress disorder and accelerate recovery from disaster-related health complications [10]. Further, youth-led movements against climate change have provided robust examples of community activism, with surveys finding Generation Z (“Gen Z”) to be more concerned about the effects of climate change than any other generation [11]. Hence, there have been growing efforts to engage youth in disaster-related volunteering, preparedness, education and response within their local communities that have yielded successful results [11]. Further, intergenerational collaboration—such as between youth and older adults—is emerging as an under-explored domain of community engagement with immense potential.

This systematic review seeks to explore the evidence for community-based interventions in addressing CDM during natural disasters, with a particular focus on solutions that empower historically excluded voices.

## Methods

### Search strategy

Our protocol was registered on PROSPERO (CRD42024573949) on August 8, 2024, which was updated on May 23, 2025, to reflect changes made to the methods upon incorporating additional reviewers. A literature review was completed using systematic methods with no date or geographic restrictions on PubMed, Google Scholar and Embase. As done with other systematic reviews contending with a high volume of Google Scholar search results [12–14], which was over 16,000 in this case, a ‘stopping-rule’ was applied [15] and only the first 100 results were screened.

Included studies were English-language, full-text research articles (primary or secondary) published in an academic journal or a scholarly research database. The goal was to derive widely applicable solutions, with an interest in historically underserved populations and intergenerational collaboration. Studies were eligible regardless of participant age or geographic location. Furthermore, as successful community interventions from old disasters (20th century and prior) may still be relevant to contemporary challenges, no date restrictions were placed on the search.

The searches were executed by one reviewer on July 28th (Google Scholar) and 29th (Embase and PubMed), 2024. Boolean operators were used to explore studies related to four concepts shaping this systematic review: (1) natural disaster, (2) chronic disease, (3) community and (4) solutions. Similar words and phrases were included for each concept. Synonyms and related terms included in the search were (1) ‘disaster’, ‘wildfire’, ‘flood’, ‘earthquake’, ‘heat wave’, ‘heat’, ‘hurricane’, ‘cyclone’, ‘storm’, ‘tornado’, (2) ‘chronic illness’, ‘chronic condition’, ‘chronic disorder’, ‘long-term illness’, ‘long-term condition’, ‘long-term disorder’, ‘non-communicable disease’, (3) ‘community partnership’, ‘partnership’, ‘intergenerational partnership’, ‘intergenerational collaboration’, ‘non-profit’, ‘collaboration’, ‘community-based’, (4) ‘strategy’, ‘strategies’, ‘action’, ‘suggestion’, ‘recommendation’ and ‘intervention’. All search terms as entered into PubMed/Embase and Google Scholar as well as wording for direct google searches are provided in S1 Text.

### Eligibility criteria and study selection

Initial search results were first title and abstract screened and then full-text screened for inclusion. The inclusion criteria were that the study must include (1) community-based solutions, which (2) must be targeted towards the management of at least one type of chronic disease (3) during at least one type of natural disaster or extreme weather event. In the scope of this review, a community-based solution was considered an intervention or recommendation that is actionable by grassroots organizations, NGOs, or volunteers without requiring upstream federal or national-level support. The inclusion and exclusion criteria, along with outcomes of interest are summarized in Table 1. Articles were eligible to pass the title and abstract screening if they met at least two of the three inclusion criteria, with the inclusion of community-based solutions required as a minimum.

**Table 1:** Eligibility Criteria.

Inclusion Criteria	Exclusion Criteria	Outcomes of Interest
• Article discusses at least one type of natural disaster or extreme weather event. • Article discusses solutions that are actionable by community members. • Article discusses at least one type of chronic disease. Article may discuss chronic diseases generally as well.	• Article does not discuss health-related community-based solutions. • Article is not in English. • Article is not published in an academic journal or scholarly database.	• Article discusses interventions involving intergenerational collaboration. • Article objectively measures the impact of a community-based solution/intervention (quantitative data) • Article addresses historically underserved groups (displaced, racial/ethnic minority and/or low-income communities).

Although studies on COVID-19–related interventions were frequently identified, the pandemic, humanitarian crises and other infectious disease outbreaks were not classified as natural disasters and were generally excluded. However, if a study met all other inclusion criteria, we conducted a full-text review to assess whether the community-based solutions it described could be applied in a natural disaster context. If they were relevant, we made exceptions to include the study. Long COVID lasting more than three months was treated as a chronic disease. If studies addressed long COVID in the context of a natural disaster and community-based solutions and all other criteria were also met, we included them.

The Preferred Reporting Items for Systematic Reviews and Meta-Analysis (PRISMA) framework [16] was used to report the systematic review, as illustrated in S1 Checklist. All title and abstract reviews along with full-text reviews were conducted by two reviewers. A third reviewer resolved discrepancies and curated the final selection of studies.

### Quality and bias assessment

The quality and risk of bias of the 46 articles included in the review was assessed using the Joanna Briggs Institute (JBI) Critical Appraisal Tools. This instrument consists of 9–11 questions answered as “yes”, “no”, “unclear” or “not applicable” [17]. As the JBI website only includes free checklists for a limited number of categories, checklists for a minority of study categories were adjusted as needed.

For this study, when most items were answered “yes”, the risk of bias was considered low and if more than two were classified as “no”, a high risk of bias was expected. All 46 studies were considered low risk and were included in the review, as none had more than two “no” answers. No scores were assigned. Assessment results are provided in S1 Data. Two reviewers conducted quality assessments for all the studies.

### Analyzing and reporting study results

A data extraction table (see S1 Table) created on Google Documents details descriptive information regarding the selected studies including first author, year of publication, title, country, methods and aims. Two reviewers read through and extracted this data from all the selected studies.

### Data synthesis

After relevant studies were selected and analyzed, it was determined that a narrative synthesis approach [18] would best represent findings, as the majority of studies were qualitative and such an approach was in line with the study goal of synthesizing evidence-based recommendations. This process involved documenting key takeaways from each study in a data extraction table and then subsequently identifying common themes across studies. If three or more studies mentioned the same theme, that theme was included as a solution category in the study. Two reviewers synthesized the findings into themes. A third reviewer resolved discrepancies and created the final, harmonized assignments of themes.

Additionally, acknowledging that the term “chronic disease” includes diverse conditions, we chose to focus on community-based solution themes that would positively impact multiple NCDs by addressing shared upstream determinants, rather than targeting individual conditions, to ensure broad applicability and feasibility of recommendations.

## Results

**Fig 1:**
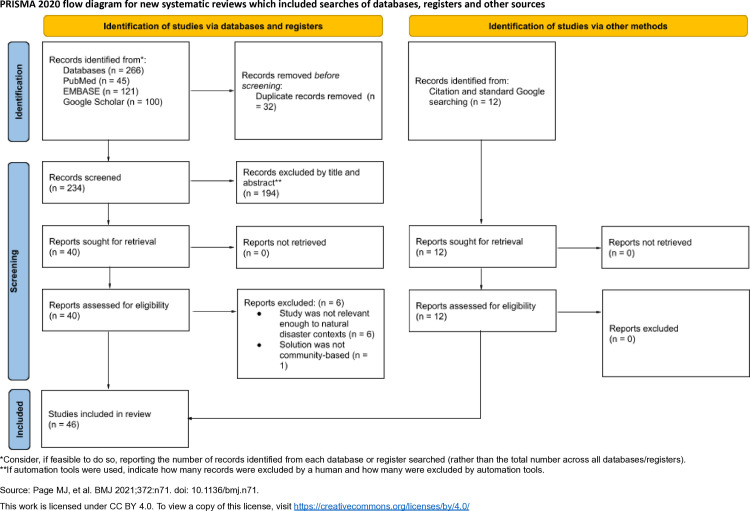
PRISMA flow diagram of study selection.

A preliminary search identified 266 studies, which were reduced to 234 after duplicates were removed using Microsoft Excel. This pool was then screened based on titles and abstracts, leaving 40 studies that met the eligibility criteria. The remaining 40 studies underwent full-text screening, resulting in 34 that met the inclusion criteria. An additional 12 studies were identified using a citation-search approach and standard Google searches, bringing the final total to 46 studies—all of which passed JBI quality appraisal. These studies were required to meet all three topical criteria and provide a thorough discussion of community-based solutions. A PRISMA flow diagram outlining the selection process is shown in Fig 1. A detailed overview of the study selection process, showing decisions and reason for exclusion for each study is shown in S2 Data.

However, during both abstract and full-text reviews, if a study did not fully meet one of the criteria (e.g., not specifically addressing chronic diseases or natural disasters), it was further evaluated to determine whether its discussion of community-based solutions would still be applicable to the topic. Six such studies that only met two out of the three inclusion criteria were included as exceptions. Corbin et al., Pickering et al. and Paudel et al. [19–21] discussed community-based solutions in the context of natural disasters but did not specifically address chronic illnesses. Their interventions, however, were broadly relevant to CDM. Plumb et al., Parmar et al., Flood et al. and Abraham et al. all focused on community-based solutions for CDM but were not studied in the context of natural disasters [22–25]. Many of the interventions discussed—such as psychosocial support, community health worker training, patient adherence to medications and self-management education—were still highly relevant to pre- and post-disaster initiatives and were therefore included.

**Fig 2:**
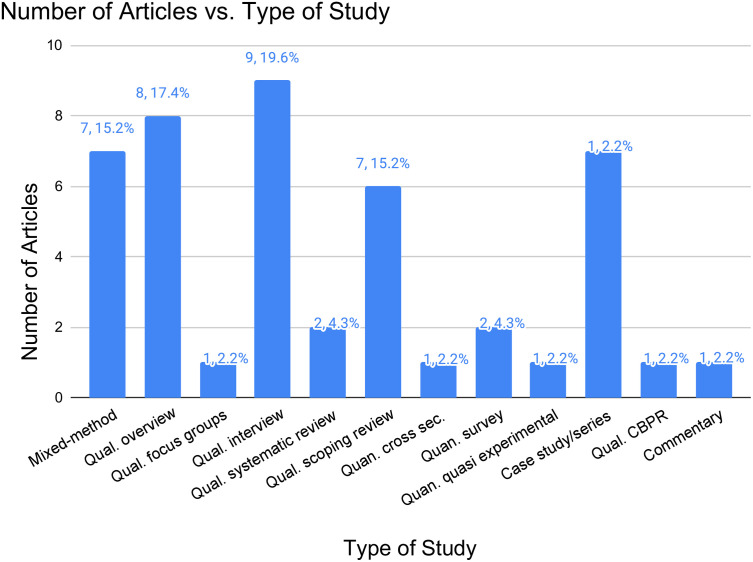
Number of articles vs. type of study bar graph.

The final included studies encompassed a variety of research designs. Quantitative studies included one quasi-experimental study, one cross-sectional study and three surveys. Qualitative studies consisted of eight overviews, two systematic reviews, six scoping reviews, eight interview studies, seven case studies/series, one community-based participatory studies, one focus group discussion and one commentary. Additionally, seven mixed method studies were included. This variation is summarized in Fig 2.

**Fig 3:**
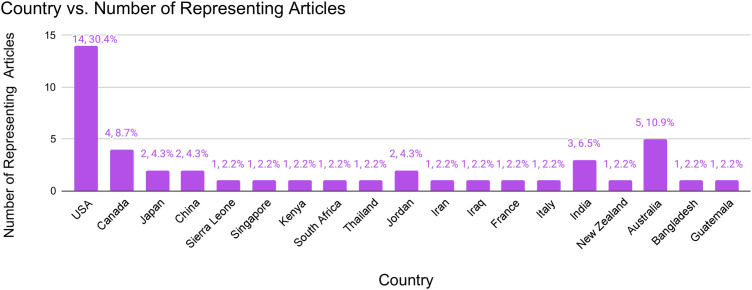
Country vs. number of representing articles bar graph.

Selected studies contained data on solutions employed in 19 countries including the USA, Canada, Japan, China, Sierra Leone, Singapore, Kenya, South Africa, Thailand, Jordan, Iran, Iraq, France, Italy, Guatemala, India, Bangladesh, New Zealand and Australia. Additional regions represented include Kashmir and multiple areas in the Caribbean. Regional representation is summarized in Fig 3. Although a majority of studies were from the US, this review also captured community-based solutions from diverse LMICs, enhancing relevance to low-resource settings that are often underrepresented in disaster research.

**Fig 4:**
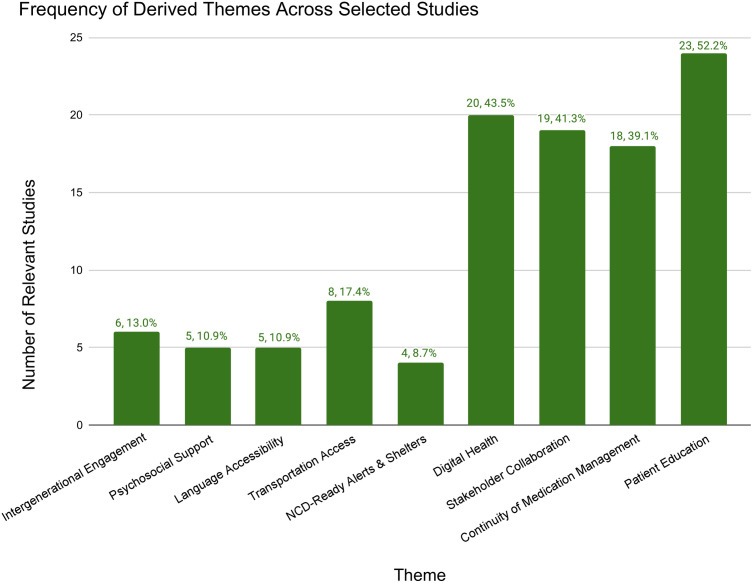
Frequency of derived themes across studies.

Themes and their corresponding solutions/interventions were derived from the 46 selected studies. If three or more studies mentioned the same type of solution, it was considered a theme. This process yielded nine themes: patient education, medication management, stakeholder collaboration, digital health, NCD-ready alerts and shelters, transportation access, language accessibility, psychosocial support and intergenerational engagement. Fig 4 details the frequency of derived themes across the selected studies, with patient education emerging as the most frequent type of solution. Table 2 summarizes a few key findings and recommendations related to the themes.

**Table 2:** Narrative Synthesis.

Theme	Findings (Successful Intervention Highlights & General Recommendations)
*Patient Education*	Extreme Heat • Hasan et al.: **Door-to-door delivery of water bottles with short messages** educating households about heat prevention was associated with increased water intake as well as reduced activity in the heat; **Informational fridge magnets, critical temperature-marked thermometers with a hotline number, colored sheets and one-on-one health education sessions** to the elderly led to: proportion of elderly people who knew whom to contact for assistance rose from 76% pre-intervention to 94% post-intervention [29]. General Suggestions • Hassan and Hou et al.: **Social media** was useful for disseminating critical information to large numbers of families with chronic diseases [4,28]. • Abraham et al.: Trained community volunteers delivered **home-based self-management kits**, **symptom monitoring sheets** and health education to India’s urban poor to establish uninterrupted care during an emergency [25]. • Ghazanchaei et al.: Risk of the exacerbation of symptoms and impacts on the health status of patients with **diabetes and chronic respiratory diseases** should be more emphasized in disaster preparedness; Federal authorities do not consider CM patients a priority and do not foster this type of awareness and health literacy, which is where community organizations need to fill the gap [41]. • Yodsuban et al.: Educational institutions and/or community organizations should design a **short course training** on the community-based flood disaster management for older adults [27].
*Continuity of Medication* *Management*	Humanitarian Crisis Settings With Natural Disasters Applications • Parmar et al.: Community health volunteers could **improve medication adherence through regular telephone check-in**. Intervention was successful in aiding a vulnerable population—**Syrian refugees** [23]. • Asgary et al. reported that having pharmacists visit Syrian refugees in Jordan to **review home medications** for NCDs significantly reduced medication errors and treatment-related issues—an approach that can be replicated through collaborations with community clinics and **trained volunteers** both before and after disaster [50]. General Suggestions: • Radhakrishnan et al., Plumb et al., Hassan et al.: Suggested community organizations **create centralized registries of patients** with chronic illnesses and available pharmacies, so that community health workers or volunteers can aid in identifying vulnerable patients and preparing their medication needs prior to disasters [48]. • Montesanti et al.: After a Canadian wildfire, forgetting health cards at home during evacuation made it difficult to refill pharmaceutical prescriptions. **Community advocacy surrounding more flexible policies** that allow patients to access their medications in shelters and receive **refills in advance** when an emergency is expected can help reduce the health deterioration of patients with chronic conditions [49].
*Stakeholder Collaboration*	Hurricane • Kopp et al.: Collaboration between the Louisiana Department of Public Health and Louisiana State University athletic facilities resulted in a **triage center** that ensured no one in Baton Rouge died from lack of dialysis treatment during the Hurricane Katrina [46]. Environmental Emergency With Natural Disaster Applications • Corbin et al.: Chlorine spill in Graniteville, South Carolina resulted in multiple fatalities and injuries due to chlorine inhalation. A **research team from the University of South Carolina collaborated with local stakeholders** to address health and environmental concerns, with critical success factors including trust-building and **engagement through town hall meetings** between the community and external responders. **Listening to the needs of affected individuals in recovery** was pivotal in the successful planning and response to the emergency; In Singapore, as a means of emergency preparedness, **local organizations invited migrants to co-host webinars and share their challenges** on social media, promoting dialogue and solutions related to about their health difficulties [19]. General Suggestions: • Arrieta et al.: Establishing **real-time information sharing systems**, mutual aid agreements and **centralized communication platforms between community organizations** can ensure timely patient evacuation, resource allocation and continuity of care. Frequent inter-agency drills and clear communication channels improve coordination, especially for vulnerable populations relying on resource-limited institutions [26].
*Digital Health*	Post-Earthquake and Tsunami • Asgary et al.: Following an earthquake and tsunami in Japan, web-based system enabled patients to monitor their blood pressure from home and communicate the data to their physicians, resulting in a **decrease in mean blood pressure from 151.3/86.9 to 129.2/70.8 over four years**; In Basra, Iraq, a system for blood glucose monitoring via a mobile app led to a **reduction in hemoglobin A1C from 8.95% to 8.05% after 7 months**. mHealth app that allowed patients to report blood pressure and glucose readings and access lifestyle counseling. Weekly educational text messages, which included behavioral modification tips for managing hypertension and diabetes, also led to **significant reductions in systolic blood pressure and hemoglobin A1C after one year** [50]. General Suggestions • Aldrich et al.: After Hurricane Charley in Florida, the CDC created population maps for the most damaged counties, which expedited the restoration of medical care and medication delivery through non-profits and community partners. **Community initiatives can adopt population mapping** (from publicly available data or partnerships with local agencies) and surveillance systems to **proactively identify high-risk areas**—such as neighborhoods with concentrations of older adults or people with chronic illnesses. This data can guide local nonprofits, community clinics and volunteers in **prioritizing outreach**, pre-positioning medical resources and coordinating care delivery [47].
*NCD-Ready Alerts &* *Shelters*	Hurricane • Schnall et al.: U.S. Virgin Islands shelter volunteers collected data on the American Red Cross Aggregate Morbidity Report form that tallies the number of client visits at a shelter’s health services every 24 hours. The study identified **volunteer shelter surveillance** as an efficient means of quickly identifying and characterizing health issues and concerns in sheltered populations following disasters, which allows for community volunteers and emergency services to quickly address them [63]. General Suggestions • Hou et al. & Hasan et al.: **Mobile text warning systems** were identified as a highly effective medium for disseminating alerts [28,29]. • Arrieta et al. 2008, Yodsuban et al., Kopp et al.: Ensuring older adults have access to emergency **shelters tailored to those with chronic conditions** was a key recommendation in three studies [26,27,46]. • Ryan et al. 2016: To maximize **shelter efficacy**, collaboration with community partners is essential to **stock commonly needed medications** for chronic disease patients [57].
*Transportation Access*	General Suggestions: • McCann et al.: Identified strategies for enhancing transportation capacity through community buses. These strategies included **contracting with bus vendors located outside the likely evacuation zone**—specifically in areas where evacuees, particularly elders, would be relocated. Local vendors were often unavailable due to their own evacuation or lack of drivers. Additionally, it is essential that these buses are **equipped to accommodate wheelchairs and necessary medical equipment**, such as oxygen tanks and pain management devices [37]. • Arrieta et al.: Underserved populations, especially those who lack personal transportation, have difficulty planning for evacuation. Furthermore, language, cultural beliefs and strong community ties such as those among the Vietnamese, Laotian and Cambodian communities of the Gulf Coast create barriers to evacuation. It is necessary to establish **linguistically and culturally sensitive transportation plans** to aid those without resources [26].
*Language Accessibility*	Migrant vulnerability • Paudel et al.: Focus group discussions and survey with Nepali migrants in Japan, identifying key recommendations. The most favored solutions included establishing **hotline services in Nepali** (62.8%), creating a crisis information hub through the Embassy of Nepal or relevant authorities (62.3%) and **publishing a guide to the Japanese healthcare system in Nepali** (41.2%) [21]. General Suggestions • Corbin et al.: Emphasized the effectiveness of communicating **disaster warnings** and preparedness strategies in **various local languages to mitigate misconceptions** [19].
*Psychosocial Support*	Earthquake • Hassan et al.: Documented a stress reduction intervention after the Haiti earthquake, which involved **controlled breathing exercises** that resulted in **significant improvements in systolic and diastolic blood pressures** among individuals with hypertension [4]. Wildfire • Montesanti et al.: Identified engagement in **cultural activities, meditative practices, sharing circles** (for discussing disaster experiences) and religious or prayer gatherings as effective coping mechanisms [49]. General Suggestions: • Nicholls et al.: Training **community health workers to serve as peer listeners and resource connectors** can provide culturally sensitive, low-barrier mental health support. These volunteers should be trained to **recognize common post-disaster psychosocial symptoms, provide informal counseling**, facilitate referrals and assist with basic needs—all of which can mitigate distress and prevent long-term mental health consequences [33].
*Intergenerational Engagement*	Extreme Heat • Hasan et al.: Long Live the Elderly“ campaign aimed at reducing heat-related mortality. Younger community members performed: **phone calls, home-care visits, organizing community events and purchasing groceries and medications** for the elderly. **13% reduction in heat-related deaths**. When introduced in France in 2006, there were **4,388 fewer heat-related deaths** than expected [29]. Broader Emergencies With Interventions Applicable to Natural Disasters • Abraham et al.: Younger and vaccinated individuals were recruited and trained to triage and deliver meals, distribute equipment (monitoring kits, symptom sheets and oxygen concentrators) and conduct home visits. This strategy aided overwhelmed healthcare facilities and **helped care for up to 10,000 residents** in Vellore [25]. General Suggestions • Pickering et al.: Community solutions should be designed to lower barriers to youth involvement in disaster relief and proposed solutions by providing **mentorship**, communicating opportunities through youth-friendly channels and providing **student recognition** for contributions [20].

### Patient education

24 studies emphasized patient education as a critical pre-disaster measure [4,23–45]. Hasan et al. described several successful heat-related education interventions, including a randomized community trial in Japan that studied the behavioral impact of door-to-door delivery of water bottles with short messages educating households about heat prevention. This intervention was associated with increased water intake as well as reduced activity in the heat. Another study in the United States supplied informational fridge magnets, critical temperature-marked thermometers with a hotline number, colored sheets and one-on-one health education sessions to the elderly [29]. The proportion of elderly people who knew whom to contact for assistance rose from 76% pre-intervention to 94% post-intervention. Hassan and Hou et al. both highlighted social media platforms as an effective means of disseminating critical information to large numbers of individuals with chronic diseases [4,28].

Interventions empowering patients in self-management have shown success in LMICs and among underserved rural populations. Flood et al. highlighted a home-based type 2 diabetes self-management intervention in rural Guatemala, which consisted of 6 home visits conducted by a diabetes educator. Following the intervention, HbA1c decreased significantly from baseline to 12 months, and improvements in systolic blood pressure along with diabetes knowledge and self-care activities were also observed [24].

### Medication continuity

Our synthesis identified 18 studies that highlighted the continuity of medication during disasters as a key priority [4,22,23,25,26,30,32,34,35,38,42,46–52]. Many studies identified community health volunteer programs and advocacy as strategies to maintain medication continuity during disasters, including among vulnerable refugee and low-income populations. Three studies suggested creating centralized registries of patients with chronic illnesses [4,22,48], which organizations can do through partnerships with community clinics, private hospitals, senior centers, assisted-living facilities and more, so that community health workers or volunteers can aid in identifying vulnerable patients and preparing their medication needs prior to disasters. Studies also proposed that community health workers provide instruction on medication management, anticipatory measures (i.e., requesting refills in advance) and resources in the event of a disaster (i.e., locations of medication distribution centers and alternate medications in case of shortages) [4]. In a study by Parmar et al., a health volunteer program was developed through a community-based, multisectoral partnership to improve secondary NCD prevention among Syrian refugees. Causal loop analysis identified that community health volunteers could improve medication adherence through regular telephone check-ins [23]. Additionally, Asgary et al. reported that having pharmacists visit Syrian refugees in Jordan to review home medications for NCDs significantly reduced medication errors and treatment-related issues—an approach that can be replicated through collaborations with community clinics and trained volunteers [50].

Furthermore, community-based advocacy is needed to create more flexible, systemwide policies to maximize patients’ ability to build and maintain prescription medication reserves during disasters [30,46,47,49,51,53]. For instance, during Hurricane Katrina, individuals who evacuated to government-sponsored emergency shelters were not allowed to bring in their home medications. This policy disproportionately impacted low-income residents, as a greater proportion of households (20.9% vs. 15.3%) lived below the poverty line in damaged areas compared to undamaged areas [54]. Yet in this emergent situation, new medications were not accessible or delivered in a timely manner [55]. Montesanti et al. also noted how after a Canadian wildfire, forgetting health cards at home during evacuation made it difficult to refill pharmaceutical prescriptions [49]. Community advocacy surrounding more flexible policies that allow patients to access their medications in shelters and receive refills in advance when an emergency is expected can help reduce the health deterioration of patients with chronic conditions [55].

### Language accessibility

Five studies identified protocol dissemination in multiple languages as a pre-disaster necessity in order to protect vulnerable populations [19,21,24,26,41]. Paudel et al. conducted focus group discussions with Nepali migrants in Japan and highlighted significant language-related barriers to disaster preparedness. From these discussions, the researchers developed a multiple-choice survey with 937 respondents, identifying key recommendations. The most favored included establishing hotline services in Nepali (62.8%), creating a crisis information hub through the Embassy of Nepal or relevant authorities (62.3%) and publishing a guide to the Japanese healthcare system in Nepali (41.2%) [21]. Similarly, Corbin et al. emphasized the effectiveness of communicating disaster warnings and preparedness strategies in various local languages to mitigate misconceptions [19]. Lastly, Flood et al’s diabetes education intervention in rural Guatemala utilized a curriculum culturally and linguistically tailored to rural Mayan populations and observed significant improvements in patient health and self-care [24]. Linguistic accessibility bolsters health equity and allows excluded or at-risk populations to be healthier and more aware amidst disasters.

### Stakeholder collaboration

Collaboration among diverse stakeholders consistently emerged as a key recommendation for optimizing community-based disaster preparedness. For example, Corbin et al. highlighted the response to a chlorine spill in Graniteville, South Carolina, which resulted in multiple fatalities and injuries due to chlorine inhalation. A research team from the University of South Carolina collaborated with local stakeholders to address health and environmental concerns, with critical success factors including trust-building and engagement through town hall meetings between the community and external responders. Listening to the needs of affected individuals during recovery was pivotal in the successful planning and response to the emergency. Another example of effective pre-disaster community collaboration is presented by Corbin et al. in their case studies on coronavirus 2019 (COVID-19) in Singapore. Local organizations invited migrants to co-host webinars and share their challenges on social media, promoting dialogue and solutions related to their health difficulties, which is applicable to natural disasters [19].

The importance of robust communication among relevant systems, workers, organizations and community members is supported by 18 additional studies [4,22,26,30,31,35,37,40,42,45–48,53,56–59]. Kopp et al. illustrate this through the aftermath of Hurricane Katrina, where pre-disaster communication and partnerships, particularly the collaboration between the Louisiana Department of Public Health and Louisiana State University athletic facilities, resulted in a triage center that ensured no one in Baton Rouge died from lack of dialysis treatment during the crisis [46]. Although both collaborators were government funded, the initiative remains actionable on a community level (volunteers and community/university clinics setting up triage spaces for chronic disease patients).

### Transportation access

Eight studies highlight the critical need for more accessible and effective evacuation transportation, particularly for historically underserved groups, who often lack consistent access to transportation for evacuation or reaching special-needs shelters [26,35,37,38,44,59–61]. For these populations, the involvement of community aid agencies and local organizations is vital in coordinating transportation before a disaster and ensuring follow-up care afterward [62].

McCann et al. identified key strategies for enhancing transportation capacity through the use of community buses. These strategies included contracting with bus vendors located outside the likely evacuation zone—specifically in areas where evacuees, particularly elders, would be relocated. Local vendors were often unavailable due to their own evacuation or lack of drivers. Additionally, it is essential that these buses are equipped to accommodate wheelchairs and necessary medical equipment, such as oxygen tanks and pain management devices [33].

Furthermore, Arrieta et al. highlighted that language, cultural beliefs and strong community ties such as those among the Vietnamese, Laotian and Cambodian communities of the Gulf Coast create barriers to evacuation. Hence, it is necessary to establish linguistically and culturally sensitive transportation plans to aid underserved populations [26].

### Digital health

Twenty studies emphasized the importance of enhancing the accessibility and availability of digital health services during disasters [4,21,27–31,35,37,40,47,48,50,51,57,58,60,63–65]. In Paudel et al.‘s study involving Nepali migrants, nearly half of the participants acknowledged the value of telehealth services in accessing healthcare within Japan and Nepal [21]. Community involvement is crucial in this context, including compiling a registry of telehealth providers, connecting patients with these providers, educating the community on how to access telehealth services and ensuring the availability of translation services for non-native speakers. Telehealth models can also be adapted to community health volunteer programs that help reach underserved communities by having volunteers engage in monthly telephone calls to provide education on self-management and psychosocial support, assess access and adherence to medication and screen for complications that required urgent referrals.

Additionally, population mapping and surveillance systems can help identify regions with a higher burden of older adults or chronic diseases, facilitating resource allocation by community organizations. Aldrich et al. noted that after Hurricane Charley in Florida, the CDC created population maps for the most damaged counties, which enabled interviews with representatives from over 600 households with older adults. This information helped local healthcare providers expedite the restoration of medical care and medication delivery through non-profits and community partners [47]. Community initiatives can adopt population mapping and surveillance systems (from publicly available data or partnerships with local agencies) to proactively identify high-risk areas—such as neighborhoods with concentrations of older adults or people with chronic illnesses. This data can guide local nonprofits, community clinics and volunteers in prioritizing outreach, pre-positioning medical resources and coordinating care delivery.

Asgary et al. detailed several successful technology-based interventions. For instance, following an earthquake and tsunami in Japan, a web-based system enabled patients to monitor their blood pressure from home and communicate the data to their physicians, resulting in a decrease in mean blood pressure from 151.3/86.9 to 129.2/70.8 over four years. Similarly, in Basra, Iraq, a system for blood glucose monitoring via a mobile app led to a reduction in hemoglobin A1C from 8.95% to 8.05% after six months. Another intervention described by Asgary et al. involved an mHealth app that allowed patients to report blood pressure and glucose readings and access lifestyle counseling. Weekly educational text messages, which included behavioral modification tips for managing hypertension and diabetes, also led to significant reductions in systolic blood pressure and hemoglobin A1C after one year [50]. Community organizations can aid clinics in transitioning to these tools or even work to make modifications to them.

### NCD-ready alerts and shelters

Four studies highlighted the efficacy and necessity of early warning systems, shelters and hotline services to assist individuals living with NCDs [29,40,42,66]. Of these, a plurality of studies focused on extreme heat, which poses a particularly higher risk of morbidity and mortality among individuals with chronic diseases. Mobile text warning systems were identified as a highly effective medium for disseminating alerts. Hasan et al. detailed multiple successful warning service interventions related to heat. For example, a heat action plan in Ahmedabad, India, which implemented a three-tiered early warning system, was associated with a significant reduction in heat-related deaths, with an estimated 2,380 deaths avoided. Additionally, the study described a manual intervention approach in Canada between 2000–2007, which focused on providing heat safety reminders and warnings via phone calls and home visits to patients in hospitals and home-care facilities. This intervention was credited with preventing 2.52 deaths per day during the 2004–2007 period [29].

Additionally, ensuring older adults have access to shelters equipped to manage chronic diseases is key. To achieve this, collaboration with community partners was additionally identified as essential to stock medications commonly used in chronic disease management. In a study following hurricanes Irma and Maria, Schnall et al. also found that shelter surveillance was an efficient means of quickly identifying and characterizing health issues and concerns in sheltered populations following disasters, which facilitated timely intervention by community volunteers and other emergency services [63].

### Psychosocial support

The necessity for stress reduction programs and psychosocial support in the aftermath of disasters was noted in five studies [4,23,27,33,49]. Hassan et al. documented a stress reduction intervention after the Haiti earthquake, which involved controlled breathing exercises that resulted in significant improvements in systolic and diastolic blood pressures among individuals with hypertension [29]. Montesanti et al. presented findings from indigenous communities in Canada following a wildfire disaster, identifying engagement in cultural activities, meditative practices, sharing circles (for discussing disaster experiences) and religious or prayer gatherings as effective coping mechanisms [49].

### Intergenerational engagement

Six studies highlighted the efficacy of intergenerational collaboration [20,25,27,29,44,57]. Youth involvement in managing NCDs during disasters was explored in three studies. Hasan et al. discussed the “Long Live the Elderly” campaign, initiated in Rome in 2004, aimed at reducing heat-related mortality. Younger community members engaged in activities such as phone calls, home-care visits, organizing community events and purchasing groceries and medications for the elderly. The program resulted in a 13% reduction in heat-related deaths compared to areas where it was not implemented. When the program was introduced in France in 2006, there were 4,388 fewer heat-related deaths than expected based on historical trends [29]. Pickering et al., in a qualitative study of youth in Ottawa, identified barriers to youth involvement in disaster relief and proposed solutions, such as providing mentorship, communicating opportunities through youth-friendly channels and recognizing their contributions [20]. Abraham et al. discussed a primary care intervention for India’s urban poor during the pandemic, outlining how younger and vaccinated individuals were successfully recruited and trained to triage and provide telephone advice on COVID-19, deliver meals, distribute equipment (monitoring kits, symptom sheets and oxygen concentrators) and conduct home visits. This strategy aided overwhelmed healthcare facilities and helped care for up to 10,000 residents in Vellore [25]. Although taken place during the pandemic, this action plan can easily be adapted and implemented as a pre- or post-natural disaster measure.

## Discussion

**Fig 5:**
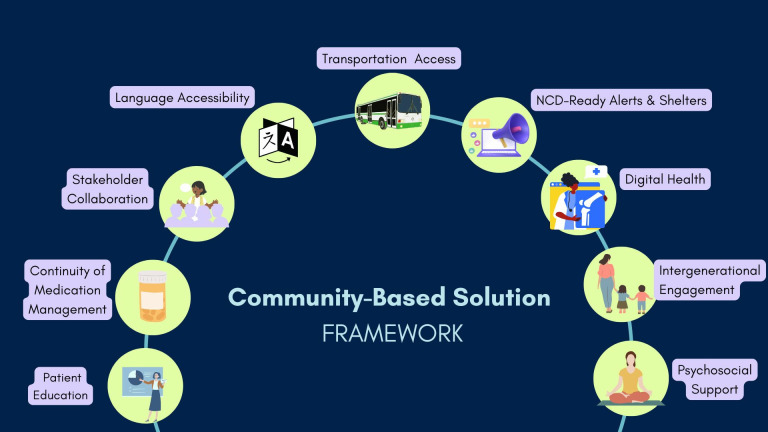
Community-based solution framework.

This review examined 46 select studies and identified 9 themes among successful community-based solutions (summarized in Fig 5): patient education, continuity of medication management, stakeholder collaboration, language accessibility, transportation access, NCD-ready alerts and shelters, digital health, psychosocial support and intergenerational engagement. This research contributes to a growing body of evidence on community-based solutions to address the health needs of people living with NCDs during natural disasters.

### Implications of findings for public health practice in the current

#### Global context.

These findings are particularly relevant in the context of climate change, under which natural disasters have been occurring with increased frequency and severity. The urgency of addressing CDM during these events is underscored by recent shifts in World Health Organization (WHO) policies. The WHO’s 2023 “Global Action Plan for the Prevention and Control of Noncommunicable Diseases” now includes disaster risk reduction as a critical component, acknowledging the compounded risks that climate change poses to individuals with chronic conditions [67]. WHO also recently developed a NCD kit — a pre-packed set of essential medicines and medical devices to meet priority NCD needs for three months in emergencies when medical facilities and regular supply has been disrupted [68].

Moreover, the COVID-19 pandemic exposed healthcare systems’ current vulnerabilities worldwide, highlighting the potential role of community-based solutions to maintain essential health services during disruptions to care. The authors’ prior research has demonstrated the value of innovative community-based solutions — incorporating visual arts, social media, culturally tailored messaging and multisectoral partnerships — to improve adherence to preventive health measures in the context of broader public health emergencies and chronic diseases like cancer [9,69]. During hurricanes Laura and Ida, Louisiana authorities struggled to evacuate residents while simultaneously minimizing the spread of COVID-19. Absence of preventive health precautions in hurricane shelters left the elderly and chronic disease patients especially vulnerable [70]. Similar health risks due to lack of disaster preparedness have been observed in other settings, such as during hurricanes Eta and Iota in Nicaragua [71]. The interventions discussed in this review specifically target these deficits, such as Abraham et al’s approach to establishing uninterrupted management of NCDs and home-based care for India’s urban poor through a community health volunteer and training program that expanded access to care and health literacy for up to 10,000 people. Further, Flood et al. discusses a community education strategy for the management of Type 2 diabetes in rural Guatemala that improved health outcomes and self-care knowledge [24], while McCann et al. provided recommendations for effective evacuation to make bus transportation more accommodating and safe for people with NCDs or other health conditions [37]. With emphasis on interventions related to education, telehealth, medication management and community health training, our findings empower community members and patients to have the resources and knowledge to better care for themselves in times of disaster and vulnerability.

These findings have clear implications for public health practice. Our review clearly highlights the ways in which the needs of patients with chronic diseases are neglected during disasters, with studies such as Arrieta et al. and Paudel et al. delineating under-recognized challenges such as interrupted medication supply chains and linguistic barriers to patient education [21,26]. With an understanding of prior successful interventions and key gaps in CDM during disasters, public health practitioners, particularly in disaster-prone regions, can begin to integrate these strategies into their disaster preparedness and response. For instance, public health officials could leverage technological approaches similar to those outlined by Aldrich et al., which details the success of population and surveillance maps in restoring access to medical care [47]. Studies such as Asgary et al. and Kopp et al. provide effective examples for health apps/monitoring systems, warning systems and maximizing stakeholder collaboration (Baton Rouge triage planning) that have yielded evidence-based improvements in health outcomes such as reduced heat and dialysis related death, less medication errors and enhanced blood pressure and A1C [46,50]. Further, the successful patient education initiatives we described in countries such as India, Guatemala, Jordan and Thailand [23,24] can serve as models for global community health volunteer programs, especially in low-resource settings. Our study found community health volunteer programs to be a notably low-cost, effective and equitable community-based solution, with one intergenerational community health volunteer program in France leading to significant reductions in heat-related death among the elderly [29]. These programs can educate patients on managing their health before, during and after disasters, provide guidance on health-specific disaster preparedness measures and enhance patient monitoring and triaging of NCD complications during crises. The solutions identified in this review highlight actionable strategies that can inform community-based disaster planning for chronic disease care.

### Recommendations for communities and NGOs

In light of these findings, we offer several recommendations for community-based solutions under different scenarios

#### Underserved communities, rural regions and LMICs.

**Training community health volunteers to foster self-management:** In settings where access to healthcare professionals, medications and stable infrastructure is limited—self-management is critical to chronic disease care. Community health volunteers can serve as crucial bridges between patients and the health system by providing basic health education, helping individuals monitor their symptoms and supporting adherence to treatment plans. In Flood et al., a program in rural Guatemala had a diabetes educator conduct home-visits, resulting in a significant decrease in hemoglobin A1c levels—demonstrating that limited, targeted support can still produce measurable health improvements [24]. These models are particularly valuable in disaster-prone or chronically underserved areas, where traditional health infrastructure may be disrupted or absent. By equipping community volunteers with simple tools and clear protocols, similar interventions can be scaled to improve continuity of care and reduce avoidable complications among NCD patients.

#### High-risk urban areas.

**Building strong networks of grassroot collaboration and utilizing surveillance to optimize care delivery:** In densely populated, high-risk urban settings, recommendations shift towards utilizing NCD-ready alerts, digital health solutions such as population mapping. Text message alerts have proven to be widely effective during urban heat waves and floods—an initiative that community organizations can adapt and tailor to critical NCD management information. Furthermore, through publicly available data or by building partnerships with local agencies, community aid organizations can use population maps and environmental surveillance data to discern where NCD burdens are highest and where the most vulnerable groups are to guide aid delivery and outreach. Lastly, strong networks of grassroot organizations established by real-time information sharing systems, mutual aid agreements and centralized communication platforms between community organizations can increase access to aid by facilitating stronger collaborations. These tools can transform chaotic, resource-limited urban disaster response into a data-informed, community-coordinated system of care.

#### Communities with language and transportation barriers.

**Accessibility and inclusivity in outreach and evacuation initiatives:** In scenarios where linguistic diversity and limited transportation options hinder both access to healthcare and evacuation, which is commonly the case for low-income households without a vehicle or countries with large numbers of migrant groups, disaster response must be linguistically and logistically tailored to ensure inclusivity and access. Local organizations should partner with cultural centers and places of worship to distribute translated materials and disaster education resources before crises occur and staff multilingual community aid hotlines with trained volunteers. Furthermore, McCann et al. highlighted that contracting transportation services from outside evacuation zones and equipping buses for patients with medical needs (e.g., oxygen tanks, wheelchairs) were critical for successful evacuation during disasters [37]. Volunteers can also provide driving services to help relocate chronic disease patients without transportation to safer shelters or assist with medication refills in advance of natural disasters.

## Strengths and limitations

This review benefits from the diverse range of study sources it incorporates. Selected studies include examples of community-based solutions from both high- and low-income countries, including the USA, Canada, Sierra Leone, Kenya, South Africa, Thailand, Japan, Jordan, Iraq, etc. This diversity enhances the applicability of our recommendations across different global settings. Moreover, this review deliberately focuses on historically underserved communities, including refugees, Indigenous populations, rural and low-income communities and migrants, which were the subjects of six key studies. Prioritizing these groups in our search and study selection processes is a critical step toward amplifying the voices of marginalized groups in climate crisis discourse.

Additionally, this review covers a broad spectrum of natural disasters—earthquakes, heat waves and floods—making the recommendations more broadly applicable across disaster contexts. The inclusion of intergenerational collaboration as a key solution is particularly beneficial, as it leverages youth engagement to strengthen disaster preparedness and CDM initiatives. Notably, our review addresses the underexplored intersection of community-based solutions, natural disasters and chronic disease management. While previous articles and systematic reviews typically address two of these areas (most often natural disasters and chronic diseases) [41,72–74], this review synthesizes and builds upon existing literature by offering actionable solutions at the community level.

This review was limited by a shortage of quantitative data measuring the impact of community-based solutions, a limitation shared by much of the public health literature on disaster response that needs to be addressed in future literature to support the efficacy of community-based action with robust data. Many of the selected studies were qualitative, utilizing focus groups or semi-structured interviews with key informants and stakeholders. While these approaches yielded valuable findings regarding how and why interventions succeeded, objective measures of impact were only available for a minority of studies. Additionally, despite the diversity of countries represented, there was an overwhelming majority of U.S-based studies in terms of quantity, which skews solutions slightly towards high-income countries. Many highly impacted regions—particularly in LMICs (e.g., Chad, Somalia, DRC, Afghanistan)—were not represented in the final sample due to a lack of peer-reviewed studies available in

English indicating a pressing data disparity in academia. Climate change has always suffered a problem of abstraction, largely due to the low representation of LMICs and communities most affected by the crisis in climate research and mainstream media. A CBC News article from 2021 describes how many scientists in the global south lack research funding and access to prestigious, expensive journals. While three-quarters of Africans live in areas experiencing climate change impacts, only 22 percent of those people live in areas with high levels of scientific research on those impacts [75].

## Conclusion

This systematic review emphasizes the intersection between the climate crisis and global burden of chronic diseases and provides evidence-based recommendations for community-level interventions. The 9 key themes identified—patient education, psychosocial support, stakeholder collaboration, language accessibility, NCD-ready alerts and shelters, continuity of medication management, digital health, transportation accessibility and intergenerational engagement—offer actionable guidance for stakeholders involved in disaster preparedness and chronic disease management.

Despite the many challenges that arise during natural disasters, including the often-overwhelming list of urgent needs, the specific care requirements of individuals with chronic diseases must not be overlooked. CDM is often deprioritized during crises, making it difficult to develop effective approaches. The solutions synthesized in this review provide a practical foundation for addressing these challenges, emphasizing scalable strategies that community members can implement with or without institutional support.

Future research should include quantitative studies that provide robust impact evaluation data, particularly in LMIC and disaster-prone settings. Specifically, longitudinal studies that track health outcomes throughout pre-disaster, during-disaster and post-disaster stages can offer meaningful insights into the efficacy of specific interventions. Moreover, research should explore the role of intergenerational collaboration in disaster preparedness, including evaluations of youth-led interventions and their impact on care continuity for vulnerable populations. Lastly, the authors acknowledge that care for chronic diseases differs vastly by condition. For example, management needs of a leukemia patient during a natural disaster differ significantly from those of a patient with hypertension or on dialysis. Hence, future studies should build on this review’s exploratory foundation to develop condition-specific, comprehensive recommendations.

Global health policymakers, NGOs and community stakeholders can utilize the findings from this review to mitigate preventable morbidity and mortality during natural disasters, especially among high-risk and underserved populations. By integrating CDM into disaster preparedness, stakeholders can ultimately help create more resilient communities for all.

## Supporting information

S1 Text Search Terms. (DOCX)

S1 Checklist PRISMA Checklist. (DOCX)

S1 Table Study Characteristics. (DOCX)

S1 Data JBI Critical Appraisal/Risk of Bias Checklist. (XLSX)

S2 Data Study Selection Process. (XLSX)
