# Supplementary material for: Community-based solutions for chronic disease management during natural disasters: A systematic review
Source: PLOS Glob Public Health. 2025 Aug 1;5(8):e0004997. doi: 10.1371/journal.pgph.0004997 (PMC12316207; doi:10.1371/journal.pgph.0004997)
Supplement: S1 Text — (DOCX) [file pgph.0004997.s001.docx]

PubMed/Embase Search Terms:

("Natural disaster" OR "disaster" OR "wildfire" OR "flood" OR "earthquake" OR "heat wave" OR "heat" OR "hurricane" OR "cyclone" OR "storm" OR "tornado") AND ("chronic disease" OR "chronic illness" OR "chronic condition" OR "chronic disorder" OR "long-term disease" OR "long-term illness" OR "long-term condition" OR "long-term disorder" OR "non-communicable disease") AND ("community" OR "community partnership" OR "partnership" OR "intergenerational" OR "non-profit" OR "collaboration" OR "community-based") AND ("solution" OR "strategy" OR "strategies" OR "action" OR "suggestion" OR "recommendation" OR "intervention")

Google Scholar:

With all of the words: Community, disaster

Exact phrase: Chronic disease

At least one of the words: Solution, strategy, recommendation, preparedness, suggestion OR lesson

Google Searches:

1. Community-based partnerships for improving chronic disease management during natural disasters
2. Community-based partnerships for chronic disease during natural disasters
3. Chronic disease during natural disasters "refugee"
