# Supplementary material for: Community-based solutions for chronic disease management during natural disasters: A systematic review
Source: PLOS Glob Public Health. 2025 Aug 1;5(8):e0004997. doi: 10.1371/journal.pgph.0004997 (PMC12316207; doi:10.1371/journal.pgph.0004997)
Supplement: S1 Table — (DOCX) [file pgph.0004997.s003.docx]

anmi

| **1st Author, Year** | **Title** | **Country (for sample or natural disaster of focus)** | **Design/Study Type** | **Theme(s)** | **Participants** | **Aim** |
| --- | --- | --- | --- | --- | --- | --- |
| Arrieta et al, 2008 | Insuring continuity of care for chronic disease patients after a disaster: key preparedness elements | USA | Qualitative, semi-structured interviews | Patient Education, Continuity of Medication Management, Stakeholder Collaboration, Transportation Access, Language Accessibility | 30 Key Informants (health and social service providers) | Provide challenges and solutions in caring for chronic disease patients after hurricanes. |
| Kopp et al, 2007 | Kidney Patient Care in Disasters: Lessons from the Hurricanes and Earthquake of 2005 | Kashmir, USA | Qualitative, Overview | Continuity of Medication Management, Stakeholder Collaboration | N/A | Delineate challenges and lessons from the Hurricanes and Earthquake of 2005 to improve kidney patient care during disasters |
| Aldrich et al, 2007 | Disaster preparedness and the chronic disease needs of vulnerable older adults. | USA | Qualitative, Overview | Continuity of Medication Management, Stakeholder Collaboration, Digital Health | N/A | Detail disaster preparedness lessons to address the chronic disease needs of vulnerable older adults |
| Hassan et al, 2020 | Management Of Chronic Noncommunicable Diseases After Natural Disasters In The Caribbean: A Scoping Review | Caribbean Region | Qualitative, Scoping Review | Continuity of Medication Management, Stakeholder Collaboration, Patient Education, Digital Health, Psychosocial Support | N/A | Suggest strategies to manage NCDs after natural disasters in the Caribbean |
| Radhakrishnan et al, 2009 | Synthesis of literature on strategies for chronic disease management post disasters | USA | Qualitative, Scoping Review | Stakeholder Collaboration, Digital Health, Continuity of Medication Management | N/A | Identify effective strategies for chronic disease management during disasters |
| Montesanti et al, 2021 | Exploring Indigenous Ways of Coping After a Wildfire Disaster in Northern Alberta, Canada | Canada | Qualitative, community-based participatory research, sharing circles, narrative review | Psychosocial Support, Continuity of Medication Management | 29 Indigenous Residents | Explore coping after wildfire disasters in Indigenous communities |
| Corbin et al, 2021 | A health promotion approach to emergency management: effective community engagement strategies from five cases | United States, Singapore, Sierra Leone, Kenya and South Africa | Qualitative, case series | Language Accessibility, Stakeholder Collaboration | N/A | Provide case examples of community engagement during disasters |
| Plumb et al, 2012 | Community-Based Partnerships for Improving Chronic Disease Management | USA | Qualitative, case series | Stakeholder Collaboration, Continuity of Medication Management | N/A | Provide case examples of community-based partnerships for chronic disease management |
| Yodsuban et al, 2021 | Community-based flood disaster management for older adults in southern of Thailand: A qualitative study | Thailand | Qualitative, mixed-methods | Digital Health, Patient Education, Intergenerational Engagement, Psychosocial Support | 127 people: Local Administration Organizations, community leaders, public sector officers, civil groups, older adult groups, and family caregivers. | Provide suggestions for community-based management of older adults in floods |
| Pickering et al, 2023 | Promoting Inclusive Institutional Culture Through Intergenerational Collaboration in Disaster Risk Reduction and Disaster Risk Management | Canada | Qualitative, semi-structured interviews | Intergenerational Engagement | 6 volunteers, 5 essential workers, and 1 participant with both essential work and volunteer experience | Suggestions to engage youth in disaster preparedness |
| Williams et al, 2018 | Evaluating Community Partnerships Addressing Community Resilience in Los Angeles, California | USA | Quantitative, social network survey | Stakeholder Collaboration | LA County Communities | Evaluate the efficacy of community partnerships for disaster response in LA |
| Asgary et al, 2022 | A systematic review of effective strategies for chronic disease management in humanitarian settings; opportunities and challenges | Japan, Jordan, USA, Iraq, | Qualitative, systematic review | Digital Health, Continuity of Medication Management | N/A | Systematically assess the evidence for interventions and identify effective strategies for the management of NCDs during disasters |
| Hou et al, 2024 | Differences in disaster warning and community engagement between families with and without members suffering from chronic Diseases: The mediating role of satisfaction with warning service | China | Quantitative, survey | Digital Health, Patient Education | 151 community leaders in Sanya; 5 households surveyed from each community, 488 responses | Understand optimal warning services for families with chronic disease members |
| Paudel et al, 2023 | Enhancing healthcare access during disasters and emergencies: Recommendations from Nepali migrants in Japan | Japan | Mixed-methods design (Focus Group Discussions, Survey) | Language Accessibility, Digital Health | 89 Nepali migrants; 937 valid survey responses | Recommend initiatives to reduce migrant vulnerability during disasters and emergencies |
| Hasan et al, 2021 | Effective Community-Based Interventions for the Prevention and Management of Heat-Related Illnesses: A Scoping Review | Canada, Italy, France, India | Qualitative, scoping review | Intergenerational Engagement, Digital Health, Patient Education, NCD-ready alerts & shelters | N/A | Detail community interventions for prevention/management of heat-related illnesses |
| Carameli et al, 2013 | Planning for chronic disease medications in disaster: perspectives from patients, physicians, pharmacists, and insurers | USA | Mixed-methods design (Focus group discussions, interviews) | Stakeholder Collaboration | Convenience sample of 158 Los Angeles County adults | Provide suggestions for chronic disease medication planning in LA county disasters |
| Chan et al, 2007 | Medical interventions following natural disasters: missing out on chronic medical needs | N/A (scoping review, countries represented not listed/synthesis not targeted to any specific country) | Qualitative, Scoping review | Patient Education, Digital Health, Continuity of Medication Management, Stakeholder Collaboration | N/A | Delineate recommended medical interventions to address chronic disease needs following natural disasters |
| Gichomo et al, 2019 | Improving Disaster Preparedness and Planning for Chronic Disease Populations | USA | Qualitative, semi-structured interviews | Stakeholder Collaboration, Patient Education, Digital Health | 15 participants; disaster planners and disaster relief workers | Provide solutions to improve disaster preparedness and planning for chronic disease populations |
| Blake et al, 2022 | “I need to have a plan in place”: Accessing medications and health treatments during a disaster for people with long-term health conditions | New Zealand | Qualitative, semi-structured interviews | Digital Health, Continuity of Medication Management | 20 participants with a long-term condition | Address solutions for access to medications and health treatments during a disaster for people with long-term health conditions. |
| Tomio et al, 2014 | Emergency and disaster preparedness for chronically ill patients: a review of recommendations | N/A (scoping review, countries represented not listed/synthesis not targeted to any specific country) | Qualitative, scoping review | Patient Education, Continuity of Medication Management | N/A | Provide a synthesis of preparedness recommendations for chronically ill patients during disasters |
| Ryan et al, 2016 | Reducing disaster exacerbated non-communicable diseases through public health infrastructure resilience: perspectives of Australian disaster service providers | Australia | Mixed-methods: Focus group discussion, interviews | Digital Health, Stakeholder Collaboration, Intergenerational Engagement | 40 disaster service providers representing 10 organizations | Understand the role disaster service providers could have in maximizing the treatment and care for people with NCDs during and after a disaster |
| Abraham et al, 2023 | Primary care for the urban poor in India during the pandemic: Uninterrupted management of non-communicable diseases and home-based care of patients with COVID-19 infection | India | Qualitative, case study | Intergenerational Engagement, Patient Education, Continuity of Medication Management | six poor communities in Vellore targeting a population of 10,000 residents | Establishing uninterrupted management of non-communicable diseases and home-based care of patients with COVID-19 |
| Nicholls et al, 2015 | The utility of community health workers in disaster preparednes, recovery, and resiliency | N/A | Qualitative, Overview | Patient Education, Psychosocial Support | N/A | The Utility of Community Health  Workers in Disaster Preparedness,  Recovery, and Resiliency |
| Icenogle et al, 2017 | Katrina’s legacy: Processes for patient disaster preparation have improved but important gaps remain | USA | Qualitative, semi-structured interviews | Continuity of Medication Management, Patient Education | 26 Key Informants in Mississippi and Alabama Gulf Coast | Delineating Processes for Patient Disaster using lessons from Hurricane Katrina |
| Ryan et al, 2018 | Ranking and prioritizing strategies for reducing mortality and morbidity from noncommunicable diseases post disaster: An Australian perspective | Australia | Mixed-method (sequential modified Delphi process) | Transportation Access, Continuity of Medication Management, Patient Education, Digital Health, Stakeholder Collaboration | people with NCDs and disaster service providers | Ranking and prioritizing strategies for reducing mortality and morbidity from noncommunicable diseases post-disaster |
| Nicholls et al, 2017 | Training community health workers to enhance disaster resilience | N/A | Qualitative, Overview | Patient Education | N/A | Providing guidelines for Training Community Health Workers to Enhance Disaster Resilience |
| Parmar et al, 2021 | Integrating community health volunteers into non-communicable disease management among Syrian refugees in Jordan: a causal loop analysis | Jordan | Qualitative, semi-structured interviews with causal loop analysis workshop | Patient Education, Psychosocial Support, Continuity of Medication Management | 20 Key informants - representatives from the Ministry of Health of Jordan, non-governmental organisations, United Nations agencies, CHVs and refugee patients | Integrating community health volunteers into non-communicable disease management among Syrian refugees in Jordan |
| McCann et al, 2011 | A review of hurricane disaster planning for the elderly | N/A | Qualitative, Overview | Patient Education, Digital Health, Transportation Access, Stakeholder Collaboration | N/A | Outlining optimal hurricane disaster planning for the elderly |
| Arrieta et al, 2009 | Providing Continuity of Care for Chronic Diseases in the Aftermath of Katrina: From Field Experience to Policy Recommendations | USA | Qualitative, semi-structured interviews | Continuity of Medication Management, Patient Education, Transportation Access | 30 health and social service providers from organizations in coastal Mississippi and Alabama | Elicit challenges and solutions in the provision of health care to those with chronic diseases after Hurricane Katrina in coastal Alabama and Mississippi. |
| Schnall et al, 2019 | Disaster-Related Surveillance Among US Virgin Islands (USVI) Shelters During the Hurricanes Irma and Maria Response | US Virgin Islands | Quantitative, cross-sectional | Digital Health | 1130 health-related client visits in shelters during | Outlining the importance of disaster-related surveillance |
| Andrade et al, 2024 | Data-Driven Interventions for an Emergency Preparedness System: A National Experience in Australia | Australia | Qualitative, case study | Digital Health | N/A | Delineating Data-Driven Interventions for an  Emergency Preparedness System |
| Subramaniam et al, 2020 | Advancing emergency preparedness for people with disabilities and chronic health conditions in the community: a scoping review | N/A | Qualitative, Scoping Review | Patient Education | N/A | Advancing emergency preparedness for people with disabilities and chronic  health conditions in the community |
| Ryan et al, 2015 | Analyzing the impact of severe tropical Cyclone Yasi on public health infrastructure and the management of noncommunicable diseases | Australia | Qualitative, case study (with narrative lit review) | Stakeholder Collaboration, Digital Health | N/A | Determine both the impact of NCDs post disaster and their impact on PHI and disaster-management systems |
| Ryan et al, 2017 | The role of environmental health in understanding and mitigating postdisaster noncommunicable diseases: The critical need for improved interdisciplinary solutions | Australia | Qualitative, Focus Group | Patient Education, Digital Health, Stakeholder Collaboration, NCD-Ready Alerts & Shelters | 55 environmental health specialists in Queensland. All participants experienced a disaster professionally | Mitigating  Postdisaster Noncommunicable  Diseases |
| Ghazanchaei et al, 2023 | Challenges in providing care for patients with chronic diseases during disasters: a qualitative study with focus on diabetes and chronic respiratory diseases in Iran | Iran | Qualitative, semi-structured interview | Patient Education, Language Accessibility | purposeful sampling; 46 patients with diabetes and chronic respiratory diseases and 36 stakeholders who were experienced and had theoretical knowledge | Explore challenges in providing healthcare services to patients with diabetes and chronic respiratory diseases during disasters in Iran. |
| Ansbro et al, 2022 | Chronic NCD care in crises: a qualitative study of global experts’ perspectives on models of care for hypertension and diabetes in humanitarian settings | N/A | Qualitative, semi-structured interviews | Stakeholder Collaboration, Transportation Access | 20 global experts (from NGOs, UN agencies, academia) | Synthesize expert opinion describing current care models for hypertension and diabetes (HTN/DM) in humanitarian settings in LMICs, to examine the gaps in delivering good quality HTN/DM care and to propose solutions to address these gaps. |
| Ochi et al, 2014 | Disaster-driven evacuation and medication loss: a systematic literature review | N/A | Qualitative, systematic review | Patient Education, Continuity of Medication Management, Stakeholder Collaboration, NCD-Ready Alerts & Shelters | N/A | identify the extent and implications of medication loss and the burden of prescription refill on medical relief teams following extreme weather events and other natural hazards. |
| Toner et al, 2017 | A community checklist for health sector resilience informed by Hurricane Sandy | USA | Qualitative, mixed-methods | Continuity of Medication Management | 67 key informants | Create a checklist that can be used by a variety of interested parties who have some role to play in disaster preparedness, response, and recovery in their own communities |
| Hossain et al, 2024 | Natural disasters, livelihood, and healthcare challenges of the people of a riverine island in Bangladesh: A mixed-method exploration | Bangladesh | Mixed-method (quantitative survey and qualitative interviews) | Patient Education | Total of 2921 participants (key informants for interviews) | Delve into the effects of these disasters on livelihood and healthcare challenges |
| Ji et al, 2023 | China's public health initiatives for climate change adaptation | China | Qualitative, case study | Digital Health, Transportation Access | N/A | Identify strategies for preserving China’s public health gains by successfully adapting to climate change |
| Brumberg et al, 2021 | Ambient air pollution: Health hazards to children | USA | Qualitative, overview | Digital Health | N/A | Review the short- and long-term health impacts of ambient air pollution on children and to examine individual, community, and policy strategies for its mitigation |
| Linares et al, 2020 | Impacts of climate change on the public health of the Mediterranean Basin population - Current situation, projections, preparedness and adaptation | Mediterranean Basin region | Qualitative, overview | Transportation Access, Patient Education, Intergenerational Engagement | N/A | provide an overview of the health risks posed by climate change in the Mediterranean region and to emphasize the urgent need for coordinated mitigation and adaptation strategies, especially for vulnerable populations. |
| Albright et al, 2009 | Organizing a strategic public health approach to addressing diabetes during disasters | USA | Qualitative, Case study | Patient Education, Stakeholder Collaboration | N/A | Refine the public health approach to addressing diabetes during disasters |
| Bush et al, 2011 | Impacts of climate change on public health in India: Future research directions | India | Qualitative, overview | NCD-Ready Alerts & Shelters | N/A | Review relevant literature and data, addressed gaps in knowledge, and identified priorities and strategies for future research in India. |
| Kingsley et al, 2019 | Commentary - Climate change, health and green space co-benefits | Canada | Commentary | Transportation Access | N/A | Provide a model of collective collaboration that aims to address complex issues, such as climate change and chronic diseases, through the common intervention of green spaces |
| Flood et al, 2017 | A Home-Based Type 2 Diabetes Self-Management Intervention in Rural Guatemala | Guatemala | Quantitative, Quasi-experimental | Patient Education, Language Accessibility | 90 participants from rural Guatemala | Evaluate a home-based DSME intervention in rural Guatemala |
